# Reversible Bradycardia and Transient Loss of Consciousness With Undiagnosed Hypothyroidism: A Case Report

**DOI:** 10.7759/cureus.94086

**Published:** 2025-10-07

**Authors:** Amir Khan, Muhammad Umair Sultan, Noor Un Nahar, Anita Rahman, Minahil Mukhtar

**Affiliations:** 1 General Internal Medicine, Northampton General Hospital, Northampton, GBR; 2 Internal Medicine, Northampton General Hospital, Northampton, GBR; 3 Acute Internal Medicine, Northampton General Hospital, Northampton, GBR

**Keywords:** angio ct, ecg (electrocardiogram), hypothyroidism and bradycardia, levothyroxine treatment, loss of conciousness

## Abstract

Hypothyroidism, or an underactive thyroid, is a common endocrine disorder characterized by insufficient production of thyroid hormones by the thyroid gland. It typically presents with non-specific symptoms and sometimes leads to serious cardiac and neurological symptoms. Due to the subtle onset, clinical recognition can be delayed. We present a case of a 62-year-old lady diagnosed with hypothyroidism presenting with bradyarrhythmia precipitating a syncopal attack. Extensive investigations were carried out to rule out a primary neurological or cardiac disorder, leading to diagnostic delays. A diagnosis of hypothyroidism is primarily established through blood tests showing an elevated thyroid-stimulating hormone (TSH) and low free thyroxine (T4) levels. Management involves long-term thyroid hormone replacement therapy, most commonly with levothyroxine, which effectively restores euthyroid status and alleviates symptoms in the majority of patients. Treating hypothyroidism in this patient led to complete symptom resolution, highlighting the importance of broadening our diagnostic approach in cases presenting with unexplained cardiovascular and neurological symptoms and emphasizes the importance of early detection and appropriate management to prevent complications.

## Introduction

Cardiovascular and thyroid hormone functions are interrelated and are regulated by intrinsic homeostatic mechanisms. Elevated levels of thyroid hormone cause hyperadrenergic states such as tachycardia, and depressed levels cause hypo-adrenergic states such as bradycardia [1]. Bradycardia is one of the manifestations of hypothyroidism, but is often overlooked, as the incidence of arrhythmia in hypothyroidism is lower when compared to hyperthyroid patients [2]. Eventually, when patients present, the initial focus of diagnosis is on conditions other than hypothyroidism [3]. It may present with nonspecific symptoms or even life-threatening events such as syncope or sudden loss of consciousness [4]. Considering a prevalence of 24% in women aged 60 years or older, investigating thyroid functions in the context of unexplained bradyarrhythmia is of high clinical relevance [5].

We present a case with an unusual presentation of hypothyroidism - a bradycardia-induced syncopal episode leading to a high-risk situation, a road traffic collision, where the focus was initially on exploring the underlying neurological disorder. We emphasize the value of thorough evaluation in such patients and picking up subtle signs like consistent bradycardia and long-standing constipation, leading to timely diagnosis and prompt management with complete resolution of symptoms.

## Case presentation

A 62-year-old English lady was found to be unresponsive in her car after slumping at the wheel and colliding with multiple vehicles. She had no recollection of the event and was noted by paramedics to have a Glasgow Coma Scale (GCS) of E1V2M5. On arrival at the emergency department, her GCS normalized. She had an early warning score (EWS) of three, which was primarily due to her heart rate of 45 bpm. The remaining observations included a respiratory rate of 18, blood pressure of 128/61 mmHg, saturation of 96% on room air, a temperature of 36.2°C, and an alert status on the Alert, Verbal, Pain, Unresponsive (AVPU) scale. 

On taking a thorough history, she had dizziness not related to posture, no symptoms of vertigo, chest pain, headache, ear discharge, tinnitus, vision changes, vomiting, or nausea. She reported no fever, rash, or weakness in any part of the body. There was no noticeable head injury. No urinary or bowel changes were reported by the patient. She mentioned that she suffered from constipation. She complained of lower back pain that started after the collision. She mentioned no recent travels and no family history of note. She denied any alcohol abuse or recreational drug use. Her past medical history included known bradycardia diagnosed during a pre-operative evaluation for knee surgery two years prior. Unfortunately, her general practitioner did not investigate bradycardia, and she was not taking any regular medications. 

On examination, there were no concerning findings apart from lacerations on the arms and tenderness on palpation of the lower back. Complete neurological evaluation and the rest of the systemic examination were unremarkable. She had a postural drop of 21 mmHg. 

The patient was admitted, and a set of baseline investigations and a CT head non-contrast was requested to rule out the possibility of a bleed. Given the unremarkable findings on the CT scan of the brain, a CT aortic arch was done to rule out posterior circulation stroke, which was also ruled out. Heading more in the direction of underlying cardiac pathology, we did a 24-hour Holter monitoring and an echocardiogram as well. Table 1 outlines her baseline blood work up. 

**Table 1:** Baseline investigations on presentation in the Accidents and Emergency department CCP: Cyclic Citrullinated Peptide; INR: International Normalized Ratio; APTT: Activated Partial Thromboplastin Time; eGFR: Estimated Glomerular Filtration Rate; HDL: High-Density Lipoprotein; SLE: Systemic Lupus Erythematosus; RA: Rheumatoid Arthritis.

Test	Result	Reference Range	Interpretation
Hb (Haemoglobin)	134 g/L	120–150 g/L	Normal
WCC (White Cell Count)	4.5 ×10⁹/L	4.0–11.0 ×10⁹/L	Normal
Platelets	215 ×10⁹/L	150–400 ×10⁹/L	Normal
CRP (C-Reactive Protein)	1 mg/L	<5 mg/L	Normal (no inflammation)
Vitamin B12	217 pmol/L	133–675 pmol/L	Normal
Folate	12.9 nmol/L	>7.0 nmol/L	Normal
Vitamin D (25-OH)	54.1 nmol/L	>50 nmol/L	Sufficient
dsDNA	<9.8 IU/mL	0–27 IU/mL	Normal (SLE unlikely)
Anti-CCP	<8 U/mL	0–17 U/mL	Normal (RA unlikely)
ESR (Erythrocyte Sedimentation Rate)	6 mm/hr	<20 mm/hr	Normal
eGFR	>90 mL/min	>90 mL/min	Normal kidney function
ANA (Antinuclear Antibody)	Negative	Negative	Normal (autoimmune disease unlikely)
INR (Clotting)	0.9	0.8–1.2	Normal
APTT (Clotting)	21 sec	25–35 sec	Slightly low (clinically insignificant)
HbA1c	34 mmol/mol	<42 mmol/mol	Normal glycemic control
Sodium (Na⁺)	141 mmol/L	135–145 mmol/L	Normal
Potassium (K⁺)	3.9 mmol/L	3.5–5.0 mmol/L	Normal
Urea	3.3 mmol/L	2.5–7.8 mmol/L	Normal
Calcium	2.26 mmol/L	2.2–2.6 mmol/L	Normal
Phosphate	1.26 mmol/L	0.8–1.5 mmol/L	Normal
Cholesterol/HDL Ratio	2.2	<4.0	Favorable lipid ratio – Low cardiovascular risk

**Figure 1:**
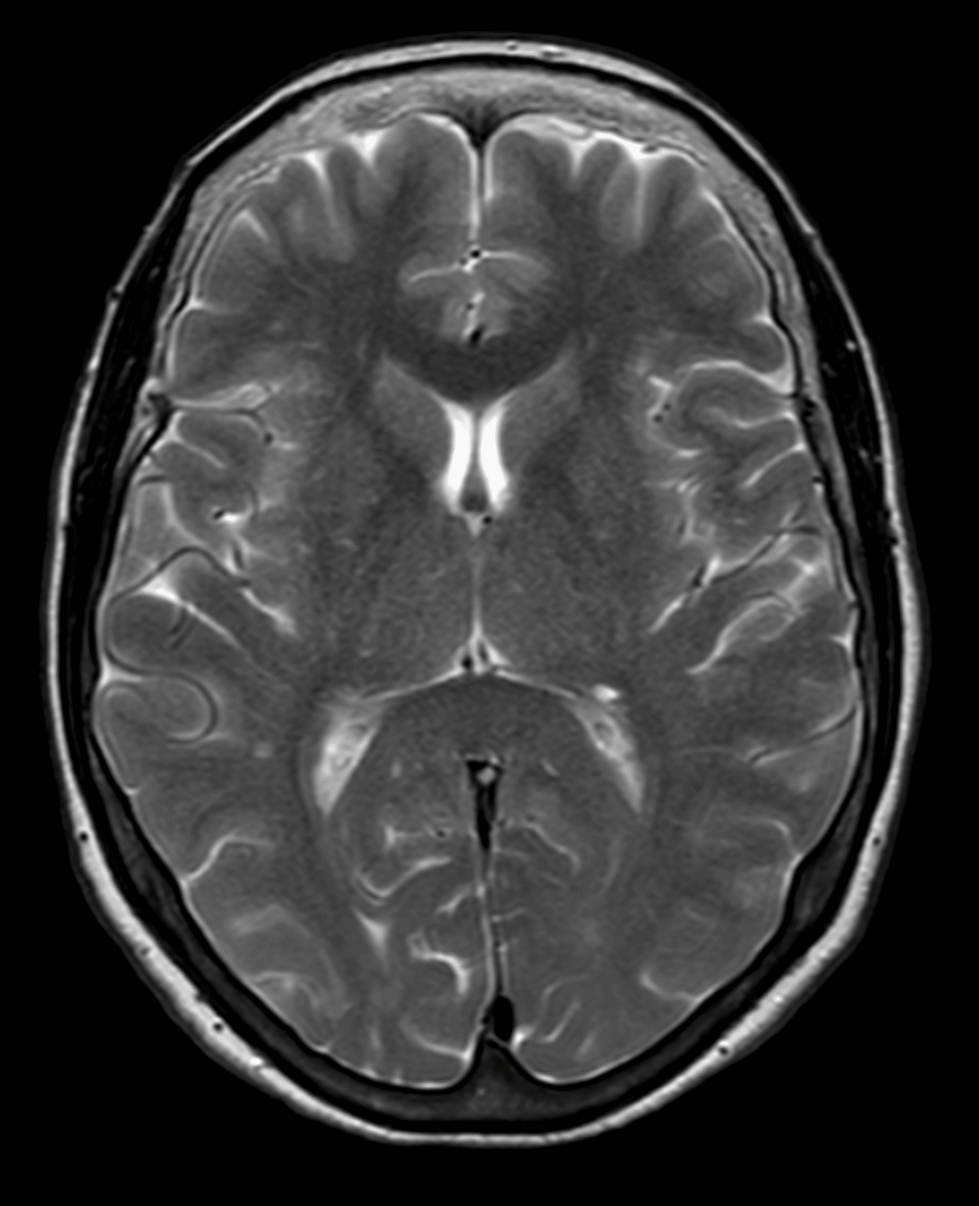
MRI brain with no evidence of an acute infarct or any other significant abnormality

Figure 1 shows the MRI brain revealing normal brain parenchyma and ruling out the possibility of a posterior circulation stroke.

**Figure 2:**
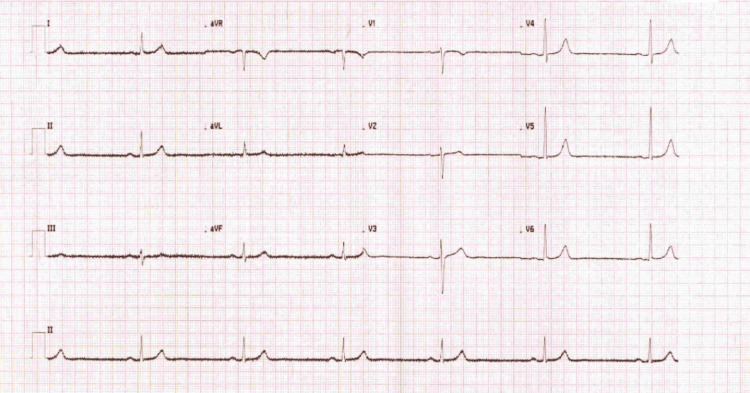
Electrocardiogram on arrival with sinus bradycardia

ECG on presentation showed sinus bradycardia with normal QRS, PR and QTc intervals (Figure 2). 

**Figure 3:**
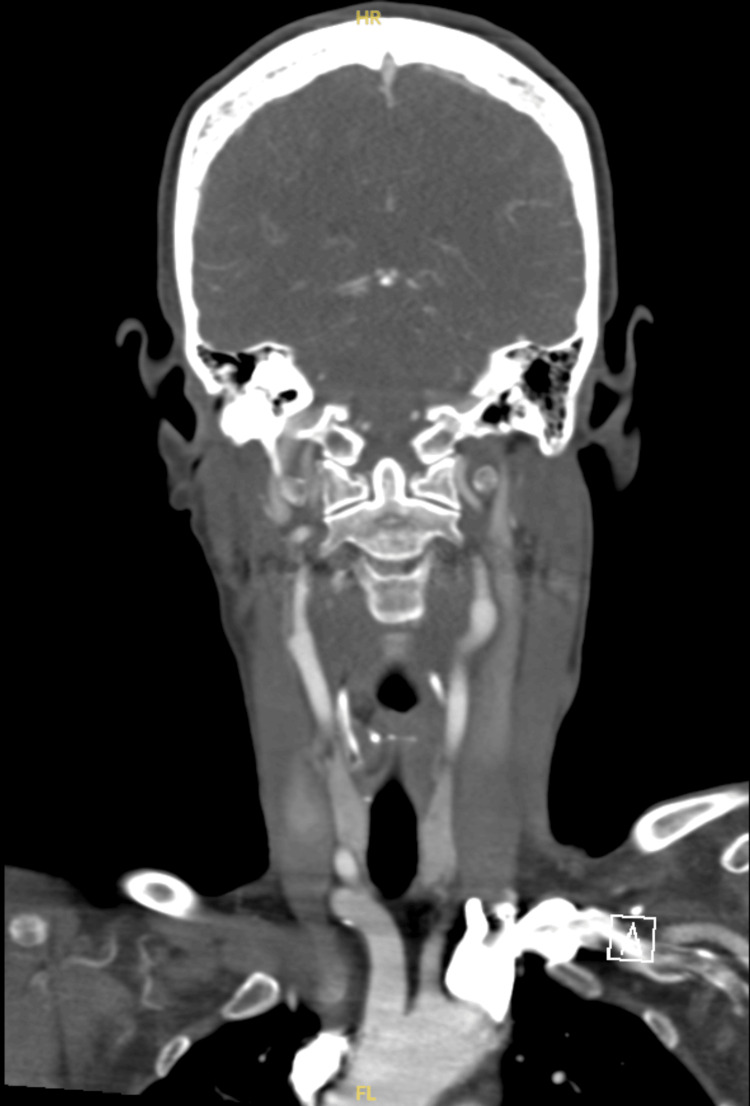
CT aortic arch showing no obvious abnormality

Figure 3 shows the image from CT aortic arch and arch vessels of normal caliber with satisfactory anterior and posterior circulation and no evidence of vascular malformation, aneurysm or stenosis.

We carried out 24-hour Holter monitoring to explore the possibility of nodal dysfunctions or underlying arrhythmogenic activity, but it was unsuccessful in finding the cause of the syncopal episode. There was sinus rhythm with persistent bradycardia. Her heart rate ranged from a maximum of 75 bpm to a minimum of 35 bpm. Findings included incidental two dropped beats with compensatory pauses post supraventricular ectopics (SVEs), and one ventricular ectopic (VE). PR and QRS intervals remained within normal limits. These findings were incidental and not potentially contributing to the presentation.

Echocardiogram ruled out structural heart abnormalities, and showed normal left ventricular size with borderline low systolic function of 54% and was otherwise unremarkable. These findings can be attributed to underlying hypothyroidism that was later diagnosed.

In view of the reassuring investigations, we further explored the bradycardia by conducting a simple thyroid hormone profile (Table 2). 

**Table 2:** Initial thyroid profile

Test	Result	Reference Range	Interpretation
TSH (Thyroid-Stimulating Hormone)	6.4 mU/L	0.4-4.0 mU/L	Elevated - Suggests hypothyroidism
Free T4 (Thyroxine)	9.2 pmol/L	10-22 pmol/L	Low - Hypothyroidism

**Figure 4:**
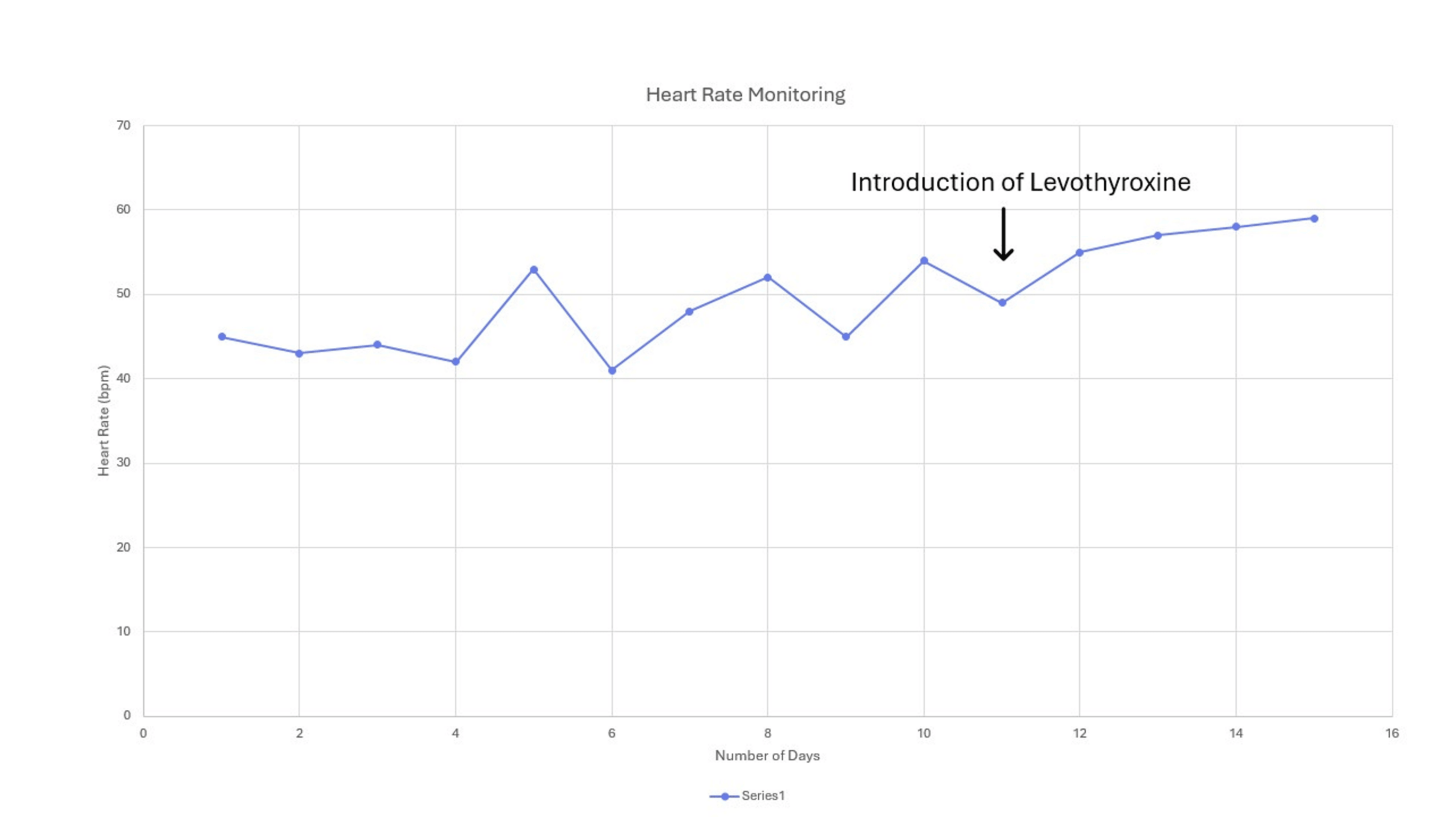
Plot of the patient's daily heart rate monitoring during the hospital stay, showing an improving trend after starting thyroxine therapy

She was started on levothyroxine 25 mcg once in the morning and monitored for heart rate. Her postural drop settled, and her heart rate improved significantly. Figure 4 shows a graph of her heart rate during admission.

**Figure 5:**
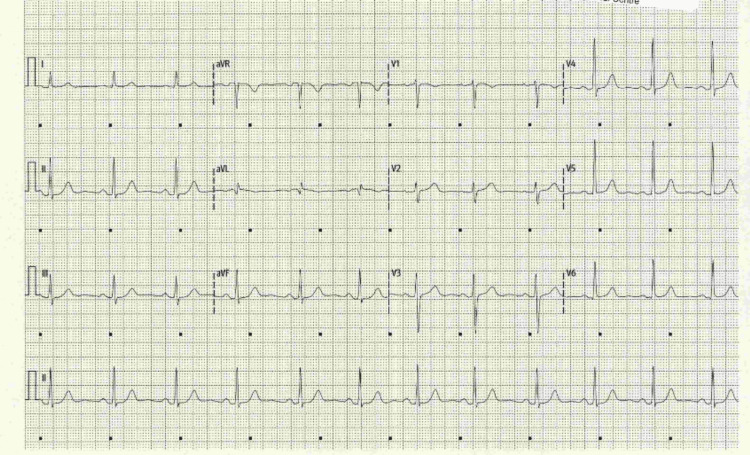
Normal sinus rhythm of the electrocardiogram at the six-week follow-up

She was referred to Endocrinology on discharge with advice and information of her medication on day 15. She was seen in six weeks time with repeat thyroid stimulating hormone (TSH) level of 1.8 mIU/L and a repeat ECG. She reported complete resolution of her symptoms and had no new concerns during this time. Figure 5 shows the normal sinus rhythm of the ECG.

She has been booked in by her General Practitioner to repeat her thyroid function tests regularly and evaluate the need for changes in the levothyroxine dose.

## Discussion

We put forward a case with a dramatic and atypical presentation of hypothyroidism manifesting as reversible bradycardia and orthostatic hypotension leading to a road traffic accident. Sinus bradycardia is a cardiac rhythm initiated from the sinus node with a rate of less than 60 bpm, diagnosed by an ECG. While most of the patients are asymptomatic, some of them present with fatigue, dizziness, light-headedness, syncope or presyncope, and cognitive impairment [6]. A study conducted in 2023 reported a 10.3% rate of bradycardia in hypothyroid patients [7]. Although it is one of the symptoms of hypothyroidism, there is no direct comparison of TSH levels to heart rate to assist in therapeutic hormonal management [1]. 

Thyroid hormones play an important role in cardiac homeostasis; hypothyroidism can lead to decreased cardiac contractility, atrial compliance, preload, and cardiac output, and increased systemic vascular resistance secondary to endothelial dysfunction [8]. There is less β-adrenergic receptor expression and decreased sensitivity to existing receptors, eventually blunting the response to increased catecholamines [9]. Both systolic and diastolic dysfunction are associated with hypothyroidism, and it can accompany arrhythmia such as premature ventricular beats, ventricular tachycardia, or even torsade de pointes [10]. Although diastolic hypertension is common in hypothyroidism, autonomic dysfunction with disruption of the baroreceptor reflex arc can cause orthostatic hypotension, which is proven to be reversible with thyroid hormone replacement [11]. Thyroid hormones, mostly the active form T3 (Triiodothyronine), play an important role in cardiovascular physiology, modulating cardiac contractile performance and contributing to electrophysiological remodeling [12]. 

When bradyarrhythmia arises, one should rule out other secondary causes such as medications, infections, hormonal imbalances, abnormalities in conduction, and neurological conditions. In our case, we ruled out infection due to a lack of fever and source; neurological factors were ruled out by scans. However, there was an imbalance in thyroid hormone levels, suggested by blood reports showing high TSH and low T4, which could lead to significant cardiac dysfunction [1] and abnormalities in conduction, as shown by the 24-hour ambulatory ECG recording. In our case, we started levothyroxine 25 mcg with planned titration to avoid rapid cardiac stimulation or precipitation of myocardial ischemia [13], in consideration of the borderline systolic function, which showed gradual improvement in heart rate and blood pressure. Hypothyroidism is one of the rare etiologies for bradycardia that shows complete recovery with treatment [14]. Few cases have been reported which showed a favorable course with complete resolution of the atrioventricular conduction abnormality [15]. With the increase in heart rate, blood flow to the brain increases, leading to an increase cerebral perfusion, preventing a syncopal attack. 

In this case, the diagnosis was delayed, as the life threatening presentation shifted the focus more so on ruling out the underlying structural, neurological, and cardiac abnormalities, which in itself is a fair approach; but we emphasize keeping broader differentials like hypothyroidism in context of persistent bradycardia, even in absence of classic systemic symptoms. The patient's bradycardia and orthostatic hypotension significantly improved after starting low dose thyroxine replacement, leading to the complete resolution of the symptoms. 

## Conclusions

This case emphasizes the importance of considering hypothyroidism in the differential diagnoses of persistent unexplained bradycardia and syncope, particularly in elderly patients. Early diagnosis and treatment with thyroid hormone replacement can lead to complete clinical recovery, preventing potential complications, including injury or fatal accidents. Going for thorough investigations is the right way to approach a syncopal or presyncope episode, but requesting a thyroid profile to rule out a potential cause of bradycardia saves time and extensive investigations, leading to quicker diagnosis and timely management of the patient. There are several cases of hypothyroidism leading to syncope or presyncope reported in the literature, signifying the need for conducting broader studies on investigating thyroid profiles in patients with bradycardia. We hope this case report will help healthcare professionals broaden their differential diagnoses before embarking on extensive investigations. 
